# Dynamic Scene Stitching Driven by Visual Cognition Model

**DOI:** 10.1155/2014/981724

**Published:** 2014-02-03

**Authors:** Li-hui Zou, Dezheng Zhang, Aziguli Wulamu

**Affiliations:** ^1^School of Computer and Communication Engineering, University of Science and Technology, Beijing 100083, China; ^2^Beijing Key Laboratory of Knowledge Engineering for Materials Science, Beijing 100083, China

## Abstract

Dynamic scene stitching still has a great challenge in maintaining the global key information without missing or deforming if multiple motion interferences exist in the image acquisition system. Object clips, motion blurs, or other synthetic defects easily occur in the final stitching image. In our research work, we proceed from human visual cognitive mechanism and construct a hybrid-saliency-based cognitive model to automatically guide the video volume stitching. The model consists of three elements of different visual stimuli, that is, intensity, edge contour, and scene depth saliencies. Combined with the manifold-based mosaicing framework, dynamic scene stitching is formulated as a cut path optimization problem in a constructed space-time graph. The cutting energy function for column width selections is defined according to the proposed visual cognition model. The optimum cut path can minimize the cognitive saliency difference throughout the whole video volume. The experimental results show that it can effectively avoid synthetic defects caused by different motion interferences and summarize the key contents of the scene without loss. The proposed method gives full play to the role of human visual cognitive mechanism for the stitching. It is of high practical value to environmental surveillance and other applications.

## 1. Introduction

Wide field of view (FOV) is demanded in many application domains, such as intelligent transportation, military defense, and civil security. A larger scope of image information is beneficial for improving the reliability and the safety of the system. However, the FOV of an ordinary camera is usually much smaller than that of humans due to the limitations of the fabrication process of enlarging the sensor size. Image stitching technology supplies an effective solution for breaking the limitation of the camera FOV, which is getting more and more attentions of researches. It is to align a sequence of overlapping images and blend the overlapping regions to form a seamless wide FOV image. The techniques nowadays can be summarized into two mainstreams: one is represented by Szeliski who proposed the classical stitching model based on geometric relationships of camera motions [1], and the other is represented by Peleg et al. who proposed an improved adaptive manifold mosaicing model [2]. The former one is to extract the geometric transform between partly overlapped adjacent images for image registration and fusion [3–5]. This model is deemed as the foundation of image alignment and stitching research, handling many camera motions, that is, translation, rotation, affine, projective motion, and so forth. The latter one is to cut narrow strips which are perpendicular to optical flow from high-overlapping images and paste their warped strips whose optical flows become parallel to the camera motion direction to form the output manifold mosaics adaptively. Such stitching model breaks through the restriction of camera motions and promotes the development of image stitching, becoming a new research focus [6–9].

Both of the above two categories of image stitching algorithms address the registration and the blending processes on pixel levels, ignoring the visual perception mechanism of humans and the relations among image contents. Sometimes the algorithms cannot guarantee the integrity of interesting contents, especially when the camera capturing platform moves and the scanned scene contains multidimensional moving objects. Some potential stitching defects, for example, object clipping, motion blurring, or ghosting, caused by moving objects and scene movement as well as parallax, easily appear in the final mosaic image. There is still a practical challenge in such dynamic scene stitching.

Image is a kind of nonstructural perception information. Comparing to direct pixel operations, how to cooperate with human cognitive mechanism and relative mathematic models to construct new computational models and methods for image processing is a meaningful and necessary work. It is helpful for improving the process efficiency and further comprehensive understanding if considering the guide role of visual cognitive mechanism as much as possible. In this paper, we address the dynamic scene stitching from the visual cognition point of view, and an effective stitching approach driven by hybrid-saliency-based visual cognition model for dynamic video sequence is proposed to avoid synthetic defects caused by different movements of the scene. It considers human visual perception mechanism first. And a cognition model is proposed, consisting of multiple visual stimuli, that is, intensity, edge contour, and scene depth saliencies of the input frames. Moreover, under the manifold mosaicing framework, the stitching process is formulated as a cut path optimization problem in a constructed space-time graph from the original input video volume. The proposed cognitive model constrains the cutting energy function for column width selections during the manifold synthesis. The effectiveness of the idea, introducing visual cognitive mechanism into the image stitching process, is verified by the experiments. The key salient contents of the wide FOV scene can be summarized without missing or deforming. The algorithm is conducive to support further analysis of global situations within the wide FOV, and it could provide a concrete reference and some inspiration to other problems in image processing driven by visual cognition as well.

The paper is organized as follows.  Section 2 formulates our dynamic scene stitching problem in mathematical descriptions.  Section 3 addresses the hybrid-saliency-based visual cognition model and its calculation details.  Section 4 gives the solutions of the output manifold via graph construction and cut path optimization at a minimum cognitive cutting cost.  Section 5 shows the comparisons and experimental results in support of the effectiveness of the proposed method. Finally, we conclude this paper in Section 6.

## 2. Problem Formulation

**Figure 1:**
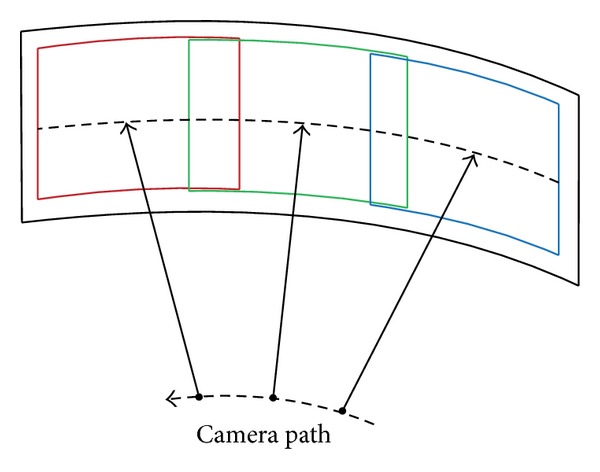
The camera path and its motion mode.

We assume that the original dynamic scenes are captured by a camera settled on a horizontal stabled pan unit which can move in a smooth path, and scanning the scene with a certain semirotation, as shown in Figure 1. The video frames of the dynamic scenes are high-overlapped in the major scanning direction and seldom vertical movements. Since manifold mosaicing algorithm is an effective solution for breaking through the restriction of camera motions, we stitch the dynamic scene in this framework. The spirit of manifold-based mosaicing technique is to cut and paste proper strips, similar to the “scanning line” in 1D linear camera imaging, into an adaptive manifold of the output mosaics. The strips from each input frame are required to be perpendicular to the optical flow and proportional to the camera motion [2].

**Figure 2:**
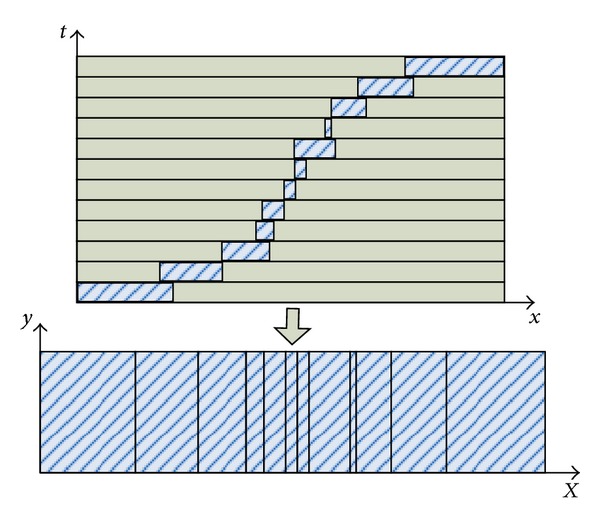
The principle for dynamic scene stitching.

Under the scheme of manifold mosaicing and inspired by [7, 8], an idea of avoiding cutting moving objects or other regions for dynamic scene stitching is to select different widths of columns from every frame to form strips and align them smoothly into an adaptive nonlinear manifold. The process can be summarized as in Figure 2. The aligned neighboring strips must look locally like the real scene without any visual artifacts.

**Definition 1**

Given a set of space-time volumes  *V*(*x*, *y*, *t*), where *V*(*x*, *t*) represents the *x*th column of the *t*th frame, the output stitching image has *L* columns in all, that is, {*θ*_*i*_ | *i* = [1, *L*]}. The mapping Γ(*θ*_*i*_) between the output column *θ*_*i*_ and the input image column *V*(*x*, Δ*y*, *t*) is the vector of (*x*, Δ*y*, *t*), in which Δ*y* is the vertical motion offset; if the major motion direction of the camera is horizontal, then Δ*y* ≈ 0. The set *P*(*x*, *t*) = {Γ(*θ*_*i*_)}_*i*=1_^*L*^ is defined as a *cut path* of the column strips along time *t*.

On the above hypothesis, each cut path corresponds to an output stitching image; therefore the process of dynamic scene stitching becomes to search and optimize the mapping relationships between the output columns and the input columns. We can then formulate the problem as the selection of the cut path at a minimum cutting cost throughout the space-time volumes.

**Definition 2**

If Γ(*θ*_*i*_) = *V*(*j*, 0, *k*) and Γ(*θ*_*i*+1_) = *V*(*g*, 0, *h*), that is, the *θ*_*i*_th and the *θ*_*i*+1_th columns of the output manifold are *V*(*j*, *k*) and *V*(*g*, *h*), that is, the *j*th column of the *k*th input frame and the *g*th column of the *h*th input frame, respectively, then the *cutting cost* is defined as follows:

(1) C(Γ,θi)=min⁡⁡{||V(j,k)−V(g−1,h)||,||V(j+1,k)−V(g,h)||}.

The cost indicates the smoothness of the transition between consecutive column strips. If *C* is sufficiently small, it implies that the appearance of these two neighboring columns, *V*(*g*, *h*) and *V*(*j*, *k*), of the output manifold is as similar as that of the columns, *V*(*g* − 1, *h*) and *V*(*g*, *h*), of the *h*th input frame, or as that of the columns, *V*(*j*, *k*) and *V*(*j* + 1, *k*), of the *k*th input frame, keeping the local consistency of the original input frames. In this paper, a hybrid-saliency-based visual cognition model is proposed and a cognitive cutting cost is designed, specifically according to the model. More computation details are introduced in the following section.

## 3. Hybrid-Saliency-Based Computational Model of Visual Cognition

Visual attention mechanism plays an important role in visual cognition [10]. How to utilize this mechanism and relative mathematic representations to establish a computational model for visual cognition and guide for image processing to improve the performance of the algorithms is a meaningful research work. In this paper, we propose a visual cognition model, considering the potential directive effects as much as possible, and apply it to dynamic scene stitching to enhance the quality of the output mosaics.

The recent research on visual psychology shows that human's attention can be caused by the visual stimulus directly or by the observation task to find specific regions which are matched to the task. Based on these two kinds of causes, the visual attention patterns can be summarized into two categories: the bottom-up pattern driven by stimulus and the top-down pattern driven by task [11]. The most general dynamic scene stitching often encounters many different kinds of image contents, such as moving cars, people or animals, artificial buildings, and nature landscape. It is hard to define a uniform task to guide the stitching. Thus, the proposed visual cognition model adopts the bottom-up way to establish the computing model. Since interesting objects usually lie in the salient regions in regard to human visual perception, the model is established based on multiple visual stimuli by forming hybrid saliency maps of the moving targets and other interesting regions. The visual cognition model mainly involves three elements of different stimuli, defined as follows:

(2) $$\begin{array}{c} \text{VCM}\left(I\right)=\alpha C_{I}\left(I\right)+\beta C_{E}\left(I\right)+\gamma C_{D}\left(I\right), \end{array}$$

where *C*_*I*_(*I*), *C*_*E*_(*I*), and *C*_*D*_(*I*) are the intensity, the edge contour, and the scene depth information of the image, respectively, and *α*, *β*, *γ* are the weight coefficients of different stimuli. These elements reflect image saliency in different aspects. The intensity is the basic representation of an image. The edge contour is another important stimulus of image contents for analysis. And using depth information to distinguish the background and the objects is the fundamental function of biological vision [12]. The composing weights can be estimated by the content-based global amplification method [13]. Driven by the visual cognition model, the overall cutting cost of the optimum output manifold along the cut path *P* becomes

(3) min⁡⁡Cost(P)=min⁡∑i=1L||CVCM(Γ,θi)||=min⁡∑i=1L−1||ΓVCM(θi)−ΓVCM(θi+1)||,

where *C*_VCM_(Γ, *θ*_*i*_) = *αC*_*I*_(Γ, *θ*_*i*_) + *βC*_*E*_(Γ, *θ*_*i*_) + *γC*_*D*_(Γ, *θ*_*i*_) indicates the saliency difference between the neighboring columns, Γ_VCM_(*θ*_*i*_) and Γ_VCM_(*θ*_*i*+1_), of the hybrid visual cognitive map volumes. The cost reveals the smoothness of the transition between consecutive column strips in intensity, contour, and salient region. The hybrid saliency differences in different visual cognition aspects are calculated as follows.

### 3.1. Intensity Cognitive Saliency Difference

Intensity is deemed as the primitive features in psychological and biological visual cognition [14] and relatively easy to compute. We describe the intensity of input images by their gray or color values directly. The intensity difference between consecutive columns, Γ(*θ*_*i*_) = *V*(*x*_*i*_, *t*_*i*_) and Γ(*θ*_*i*+1_) = *V*(*x*_*j*_, *t*_*j*_), of the input volumes, is computed as follows:

(4) CI(Γ,θi)=min⁡⁡{||Vgray(xi,ti)−Vgray(xj−1,tj)||,    ||Vgray(xi+1,ti)−Vgray(xj,tj)||},

where *V*_gray_(*x*_*i*_, *t*_*i*_) and *V*_gray_(*x*_*j*_, *t*_*j*_) are the gray values of the *x*_*i*_th and the *x*_*j*_th columns in the *t*_*i*_th and the *t*_*j*_th frames, respectively. Minimizing this difference will maintain the basic visual appearance information among the neighboring strips. It is the primary premise to keep a smooth transition.

### 3.2. Edge Contour Cognitive Saliency Difference

Besides the salient intensity feature, the geometric contour structure is also a significant factor, impacting the continuity of the mosaic strips. The saliency of contour structures can be extracted by edge detectors, for example, Sobel, Canny, and so forth, which are easy to calculate and of definite physical meanings. Nevertheless, the detection results usually depend on the extent of luminance variance and contrast changes. We suggest extracting phase congruency (PC) which reflects the behavior in the frequency domain to express the saliency of contour structure of the image. Based on many physiological and psychophysical evidences [15, 16], it is demonstrated that PC theory can provide a biologically plausible model for how human visual systems detect and identify features in an image. Compared with gradient-based edge detectors, it is not only invariant to illumination and contrast, but also superior in detecting and identifying multiple edge saliencies, including ramp edge, step edge, roof edge, and line edge. It can be considered as a dimensionless measure for the significance of a local structure. This property ensures that the PC-based contour saliency difference reflects the structural continuity cost among consecutive strips conforming to visual cognition behaviors. Therefore, the edge contour cognitive saliency difference between neighboring columns, Γ(*θ*_*i*_) = *V*(*x*_*i*_, *t*_*i*_) and Γ(*θ*_*i*+1_) = *V*(*x*_*j*_, *t*_*j*_), of the input volumes, is defined as follows:

(5) CE(Γ,θi)=min⁡⁡{||VPC(xi,ti)−VPC(xj−1,tj)||,     ||VPC(xi+1,ti)−VPC(xj,tj)||},

where *V*_PC_(*x*_*i*_, *t*_*i*_) and *V*_PC_(*x*_*j*_, *t*_*j*_) are the phase congruency values of the *x*_*i*_th and the *x*_*j*_th columns in the *t*_*i*_th and the *t*_*j*_th PC maps, respectively.

The PC map volume *V*_PC_(*x*, *y*, *t*) can be calculated from the input space-time volume *V*(*x*, *y*, *t*). Rather than defining the saliency of edge features directly at points with sharp changes in intensity, the PC model postulates that features are perceived at points where the Fourier components are maximal in phase according to the psychophysical effects on human visual perception. It is derived from the local energy model [17], a salient feature measurement in frequency domain, and initially expressed as follows:

(6) $$\begin{array}{c} \text{PC}\left(x\right)=\frac{\left|E\left(x\right)\right|}{\sum_{n}A_{n}\left(x\right)}, \end{array}$$

where *A*_*n*_(*x*) is the amplitude of Fourier components at the location *x* in the signal and |*E*(*x*)| is the local energy. The essence of the PC is to measure the phase similarity among all Fourier components. It is valued from 1 to 0, representing the saliency of features from significant down to none. However, this measure of PC does not provide good localization and it is also sensitive to noise. We adopt the improved PC based on banks of Log-Gabor wavelets and quadrature pairs of filters, which is developed by Kovesi [18] and widely used in the literature, to calculate *V*_PC_(*x*, *y*, *t*). Since the local phase obtained by Log-Gabor wavelets lacks rotational invariance, orientation samplings are required to guarantee that the salient features are treated equally at all the possible orientations. The phase congruency at position (*x*, *y*) becomes

(7) $$\begin{array}{c} {\text{PC}}_{2}\left(x,y\right)=\frac{\sum_{o}\sum_{n}W_{o}\left(x,y\right)\left\lfloor A_{no}\left(x,y\right)\Delta \Phi_{no}\left(x,y\right)-T_{o}\right\rfloor }{\sum_{o}\sum_{n}A_{no}\left(x,y\right)+\varepsilon }. \end{array}$$

The symbols ⌊·⌋ denote that the enclosed quantity is equal to itself when its value is positive and zero otherwise. *ε* is a small constant to avoid division by zero. *W*_*o*_(*x*, *y*) is a factor weight for frequency spread in orientation *o*. *T*_*o*_ is the noise threshold, the estimated noise influence. Only energy values that exceed *T*_*o*_ are counted in the result. *A*_*no*_(*x*) is the local amplitude of frequency component on scale *n* and in orientation *o*. ΔΦ_*no*_(*x*, *y*) is the phase derivation function which can be expanded as follows:

(8) ΔΦno(x,y)=cos⁡⁡(ϕno(x,y)−ϕ−o(x,y)) −|sin⁡(ϕno(x,y)−ϕ−o(x,y))|,

where ${\overset{-}{\phi }}_{o}(x,y)$ is the local mean phase angle in orientation *o*. The product of *A*_*no*_(*x*) and ΔΦ_*no*_(*x*, *y*) can be calculated as follows:

(9) Ano(x,y)ΔΦno(x,y)  =eno(x,y)ϕe(x,y)+ono(x,y)ϕo(x,y)   −|eno(x,y)ϕo(x,y)+ono(x,y)ϕe(x,y)|,

where *ϕ*_*e*_(*x*, *y*) = ∑_*n*_ *e*_*no*_(*x*, *y*)/*E*(*x*, *y*), *ϕ*_*o*_(*x*, *y*) = ∑_*n*_ *o*_*no*_(*x*, *y*)/*E*(*x*, *y*). The local energy *E*(*x*, *y*) is defined as follows:

(10) $$\begin{array}{c} E\left(x,y\right)=\sqrt{{\left(\sum_{n}e_{no}\left(x,y\right)\right)}^{2}+{\left(\sum_{n}o_{no}\left(x,y\right)\right)}^{2}}, \end{array}$$

where *e*_*no*_(*x*, *y*) = *V*(*x*, *y*)∗*M*_*no*_^*e*^ and *o*_*no*_(*x*, *y*) = *V*(*x*, *y*)∗*M*_*no*_^*o*^ are the convolution results of the input image signal *V*(*x*, *y*) with even- and odd-symmetric Log-Gabor filters, *M*_*no*_^*o*^ and *M*_*no*_^*e*^, on scale *n* and in orientation *o*.

### 3.3. Scene-Depth Cognitive Saliency Difference

Depth is an important component channel in biological vision organisms. It assists in focusing attention on important locations and objects of the viewed scene. Since the human visual system has evolved predominantly in natural 3D environments, it is inspired to utilize depth information to accomplish visual task by instinct. There have been several efforts to include the depth channel in computational attention models to make the artificial visual attention biologically plausible [12, 19, 20]. In this paper, we take advantage of the characteristic that depth information has prominent effect on highlighting regional objects to define the scene-depth-based cognitive saliency difference between neighboring output columns, Γ(*θ*_*i*_) = *V*(*x*_*i*_, *t*_*i*_) and Γ(*θ*_*i*+1_) = *V*(*x*_*j*_, *t*_*j*_), as follows:

(11) CD(Γ,θi)=min⁡⁡{||Vdepth(xi,ti)−Vdepth(xj−1,tj)||,   ||Vdepth(xi+1,ti)−Vdepth(xj,tj)||},

where *V*_depth_(*x*_*i*_, *t*_*i*_) and *V*_depth_(*x*_*j*_, *t*_*j*_) are the estimated depth values of the *x*_*i*_th and the *x*_*j*_th columns in the *t*_*i*_th and the *t*_*j*_th depth label maps, respectively. The depth saliency difference reflects the regional homogeneity in visual cognition.

**Figure 3:**
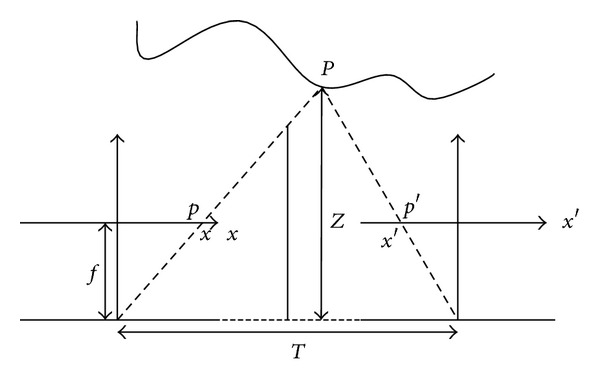
Relationship between depth and disparity.

Computing depth for an attention system is usually solved in stereo vision problems. In general, sensing the same scene from different view points, the depth information can be obtained by computing the disparity, that is, the parallax, between corresponding pixel pairs based on the triangulation principle [21]. The relationship between depth and disparity can be explained briefly as shown in Figure 3. Suppose that two corresponding projected pixels of the scene point *P*, whose depth is *Z* in the neighboring frames, are *p*(*x*, *y*) and *p*′(*x*, *y*), lying in the equal scanning line, that is, Δ*y* ≈ 0; then the disparity becomes *d*(*x*, *y*) = *x* − *x*′. If given the focal length *f*, according to the similar triangle principle, we have

(12) $$\begin{array}{c} \frac{Z}{T}=\frac{f}{d}. \end{array}$$

It shows that if given the depth *Z* of a fixed point, then the disparity between its corresponding projected pixels is determined. Conversely, the depth can be also calculated by the disparity. Based on this fundamental correspondence, they are easy to interconvert with each other. With the increase of depth, the disparity goes down to 0 at the infinite points, whereas the nearest point with maximum disparity is denoted as *D*_max⁡_. Thus, the disparity range of arbitrary points in the scene is *D*_*s*_ = [0, *D*_max⁡_], usually discretized as *D*_*s*_ = {0 = *d*_0_ < *d*_1_ < ⋯<*d*_*n*_ = *D*_max⁡_} in pixels. According to the above correspondence relationship, the depth map volume *V*_depth_ can be computed between each two neighboring frames from the disparity field, *d*_*p*_ ∈ *D*_*s*_, by matching one to a reference one and mapping to the discrete disparity space to obtain the disparity of every pixel in the reference frame. In this paper, we simplify the disparity estimation algorithm of [22] and calculate the depth cognitive saliency difference based on mean-shift disparity filter, assuming that disparity values vary smoothly in homogeneous regions and depth discontinuities only occur on region boundaries. The specific steps for depth map calculation are as follows.

*Step 1* (segment the homogeneous regions by mean-shift). We adopt mean-shift algorithm to decompose the reference frame into regions of homogeneous color or grayscale. It is easy to oversegment a whole region into multiple regions, which is preferred here to satisfy the disparity variance assumption in practice.

*Step 2* (compute the local match cost in a bidirectional way). Taking each pair of neighboring frames as the reference image and the matched image, the match cost of pixel (*x*, *y*) and disparity *d* between the reference frame and the matched frame in a local window *N*(*x*, *y*) are calculated in a bidirectional way. Consider

(13) CD(x,y,d)=(1−ω)∗CSAD(x,y,d) + ω∗CGRAD(x,y,d),CSAD(x,y,d)=∑(i,j)∈N(x,y)|I1(i,j)−I2(i+d,j)|,CGRAD(x,y,d)=∑(i,j)∈Nx(x,y)|∇xI1(i,j)−∇xI2(i+d,j)| +∑(i,j)∈Ny(x,y)|∇yI1(i,j)−∇yI2(i+d,j)|.

The matching criterion *C*_*D*_ combines sum of absolute differences (SAD) and gradient absolute differences (GRAD). It is adaptive to the scene changes and would provide better accuracy, especially on the surface with textures.

*Step 3* (estimate initial disparity map via cross-checking and WTA). In order to detect unreliable matches, a cross-checking procedure to the bidirectional matching cost is employed in conjunction with the winner-take-all (WTA) optimization strategy (choosing the disparity with the lowest matching cost). If given the range of disparities, *R*_*d*_ = [*d*_min⁡_, *d*_max⁡_], in which the number of discrete disparities becomes *N*_*d*_ = *d*_max⁡_ − *d*_min⁡_ + 1, then the initial matched disparity of the reference frame is

(14) $$\begin{array}{c} D_{\mathrm{int}}\left(x,y\right)=\arg\underset{d\in R_{d}}{\min}\;C_{D}\left(x,y,d\right). \end{array}$$

*Step 4* (simplify the computing by filtering the disparity map based on mean-shift segments). On the assumption that disparity values vary smoothly in homogeneous regions and depth discontinuities only occur on region boundaries, a single depth value is computed for each homogeneous region. The initial disparity map is filtered by taking the median disparity value of each mean-shift segment as its whole parallax, that is,

(15) $$\begin{array}{c} D_{s_{i}}=\text{median}\left(D_{\mathrm{int}}\left(x,y\right)\right),\;\left(x,y\right)\in {\text{Seg}}_{i}. \end{array}$$

After the above disparity calculation steps, the depth information is obtained indirectly. We can transform the disparity map volume into its depth map volume at last.

## 4. Cut Path Optimization via Graph Construction

**Figure 4:**
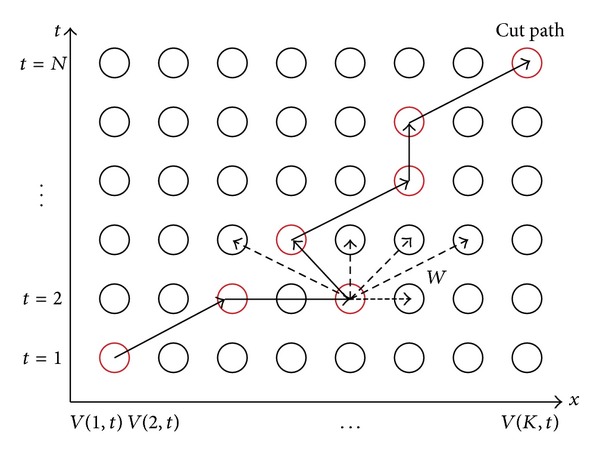
Graph construction in *x*-*t* space and its cut path for dynamic scene stitching.

Since the columns of input frames are deemed as the basic elements for forming the output manifold, every column of the input images is regarded as a node so that the video volume can be abstracted as a graph. Let *G*(*V*, *E*, *W*) denote the graph, as shown in Figure 4. The nodes *V* = {*V*(*k*, *t*)} are the *K* × *N* image columns. The edges *E* encode the possible transitions among the columns. And each edge has an associated transition cost *W* = *C*_VCM_(Γ, *θ*_*i*_), that is, the cutting cost, defined as the above cognitive saliency difference from the hybrid visual cognitive map volumes.

During the cost computation, due to the instability of point-to-point comparison of columns, we compute the hybrid cognitive saliency difference between Γ(*θ*_*i*_) and Γ(*θ*_*i*+1_) in their centered rectangle windows. Moreover, there is no need to add all possible edges to the graph since the frames come from high-overlapping video sequences. Only those edges among nearby patches, as those dashed edges in Figure 4, are computed depending on the expected maximal motion velocity.

The goal of cut path optimization is to find a shortest path from *V*_start_ to *V*_end_. It minimizes the cutting cost along the cut path, in which the salient regions are kept with minimum deformation as much as possible. Due to the efficiency of Dijkstra algorithm [23] for solving the shortest path problem between given nodes in a graph with nonnegative edge costs, we adopt it to search the cut path from the starting frame to the ending frame. Suppose *u*_0_ = *V*_start_ is the source node and *v*_0_ = *V*_end_ is the destination node. The basic idea of the algorithm is to calculate the shortest path and distance from *u*_0_ to all the possible transition nodes of *G*, in the order of their distance to *u*_0_. It stops until *v*_0_ or covering all the possible transition nodes of *G*. In the meantime, labels are used to avoid repeating and keep the computing information of every step. The algorithm steps are as follows.

**Step 1**

Set *l*(*u*_0_) = 0, and *l*(*v*) = *∞*, *S*_0_ = {*u*_0_}, *i* = 0 for *v* ≠ *u*_0_. If |*V*| = 1, then stop; otherwise go to Step 2.

**Step 2**

For each *v* in *V*∖*S*_*i*_, that is, $v\in {\overset{-}{S}}_{i}\;\;({\overset{-}{S}}_{i}=V\setminus S_{i})$, replace *l*(*v*) by min⁡_*u*∈*S*_*i*__{*l*(*v*), *l*(*u*) + *w*(*uv*)}. When *v* ≠ *u*, *w*(*uv*) = *∞*. If *l*(*v*) is replaced, put a label (*l*(*v*), *u*_*i*_) on *v*.

**Step 3**

Compute min⁡_*u*∈*S*_*i*__{*l*(*v*)}, $v\in {\overset{-}{S}}_{i}\;\;({\overset{-}{S}}_{i}=V\setminus S_{i})$, and denote the corresponding node as *u*_*i*+1_; then let *S*_*i*+1_ = *S*_*i*_ ∪ {*u*_*i*+1_}.

**Step 4**

If *i* = |*V*| − 1, then stop. If *i* < |*V*| − 1, then replace *i* by *i* + 1 and go to Step 2.

After optimizing the cut path of the dynamic volume, select the column strips in every frame according to the path and paste them together into a large adaptive manifold. The output stitching scene is then composed without deformations or other artificial defects.

## 5. Experiments and Comparisons

In order to testify the performance of the proposed algorithm in dealing with moving objects, we captured a series of video sequences under the previously described camera motion mode. Different complex human movements were involved in the videos. And the proposed method was also compared to another two manifold mosaicing algorithms [6, 8].

**Figure 5:**
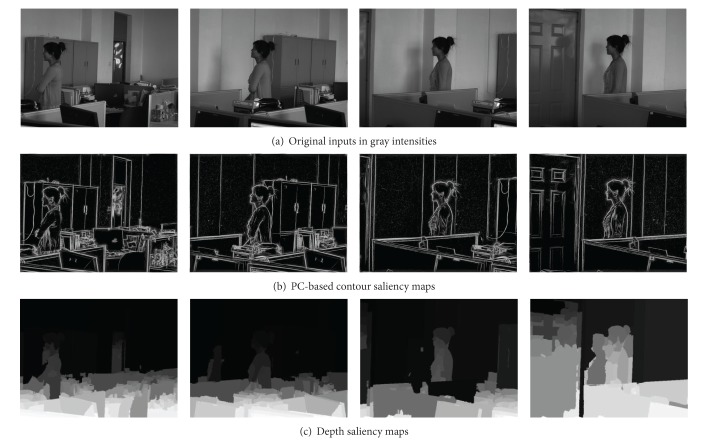
Multiple visual stimuli of dynamic scene 1.

**Figure 6:**
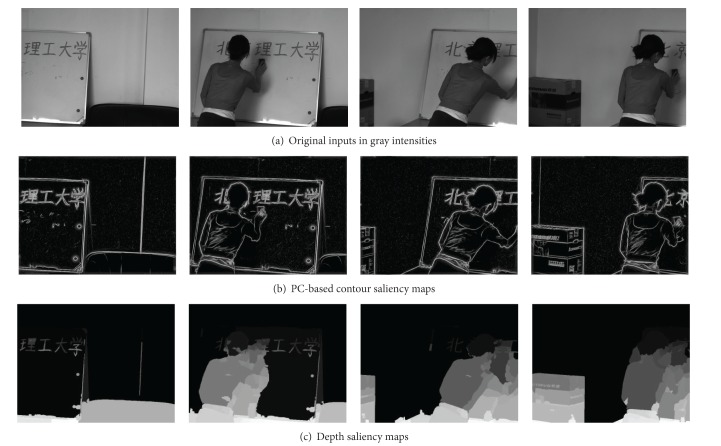
Multiple visual stimuli of dynamic scene 2.

**Figure 7:**
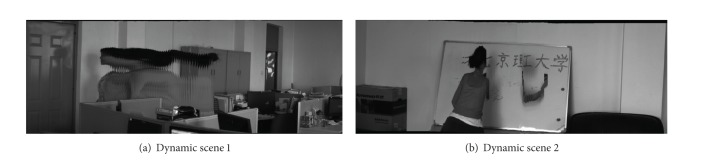
Stitching results of [6].

**Figure 8:**
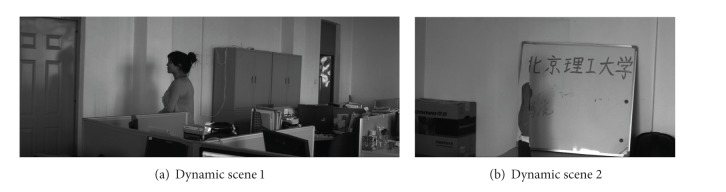
Stitching results of [8].

**Figure 9:**
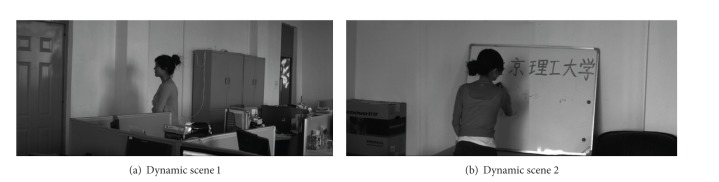
Stitching results of the proposed method.

**Figure 10:**
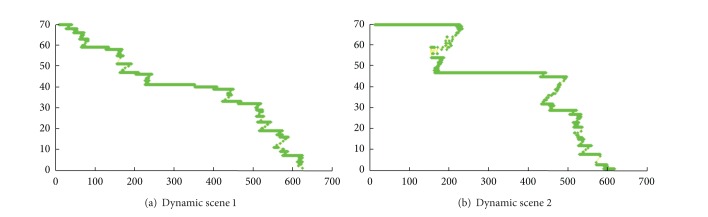
Cut paths for forming the optimum output manifolds.

Two typical examples of dynamic scene stitching are shown in Figures 5 and 6. The visual cognitive maps of different salient stimuli are seen in Figures 5 and 6, in which (a)s are original inputs in gray intensities, (b)s are the PC-based contour saliency maps, and (c)s are the depth saliency maps. The stitching results of [6, 8] and our proposed algorithm are shown in Figures 7–9, respectively. Reference [6] estimates the global motion parameters between neighbor frames at first by iterating Lucas-Kanade optical flow under Gaussian pyramid strategy. And then the strips are selected from the middle of video frames according to the classical manifold mosaicing technique. The final output mosaics are composed without any a priori perception or optimization. The algorithm of [6] can stitch the background of the scene entirely, but it is poor in dealing with nonrigid moving objects, as seen in Figure 7. Instead of treating scene stitching as geometrical alignment, [8] poses it as a minimal appearance distortion in pure pixel processing level. The algorithm of [8] shows some effectiveness on maintaining moderate moving objects during the stitching, as the walking person in scene 1 is only elongated a little bit; see Figure 8(a). Nevertheless, when the scene contains more complex movements, such as the movements of the person, cleaning the blackboard, in scene 2, the moving object would be easily clipped, as seen in Figure 8(b). The performance of the algorithm in [8] needs to be improved. The stitching results of the proposed method are shown in Figure 9. And the corresponding cut paths are shown in Figure 10. It can be seen that, driven by the visual cognition model, the cut paths successfully avoid cutting the salient movement regions and the backgrounds as well, whereas the other two algorithms cannot guarantee the integrity of the moving objects in different degree, especially when the object moves in high mobility, since their manifold synthesis processes neglect the visual cognitive mechanism stimulated by multichannel saliencies.

After a sequence of experimental tests, it shows that the proposed method is robust to different dynamic movements. The hybrid-saliency-based cognitive model guarantees the stitching effect nicely. The proposed method can solve the dynamic scene stitching problem effectively.

## 6. Conclusions

This paper investigates dynamic video sequence stitching, especially under the situation that the scene, captured on a movable platform, contains moving objects or other important interesting regions. There is a great challenge to preserve the moving objects and the salient regions in the final stitching image as their original looks without any missing or deformation. In our research work, we proceed from human visual cognitive mechanism and analyze multiple visual stimuli to construct a hybrid-saliency-based cognitive model. Constrained by this model and combined with the manifold mosaicing framework, we proposed an effective dynamic scene stitching algorithm without any camera calibration and motion estimation. It can give full play to the role of visual cognitive mechanism of human in image synthesis for global scenes and reasonably avoid synthetic defects, such as motion blur and object clipping. The experimental results show that the proposed method performed quite well. It can be applied to wide-field monitoring system for supporting global situation judgments and decision-making, or other security investigations. The next goal is to study the sensitivity towards the selection of parameters in the cognition model, cooperating with quantitative stitching assessment.

## References

[1] Szeliski R (1996). Video mosaics for virtual environments. *IEEE Computer Graphics and Applications*.

[2] Peleg S, Rousso B, Rav-Acha A, Zomet A (2000). Mosaicing on adaptive manifolds. *IEEE Transactions on Pattern Analysis and Machine Intelligence*.

[3] Zou L, Chen J, Zhang J, Lu J (2012). Image mosaicing algorithm for dynamic scenes using multi-scaled PHOG feature and optimal seam. *Pattern Recognition and Artificial Intelligence*.

[4] Jia J, Tang C-K (2008). Image stitching using structure deformation. *IEEE Transactions on Pattern Analysis and Machine Intelligence*.

[5] Zhao ZS, Feng X, Teng SH (2012). Multiscale point correspondence using feature distribution and frequency domain alignment. *Mathematical Problems in Engineering*.

[6] Zou L-H, Chen J, Zhang J, Dou L-H An image mosaicing approach for video sequences based on space-time manifolds.

[7] Rav-Acha A, Engel G, Peleg S (2008). Minimal Aspect Distortion (MAD) mosaicing of long scenes. *International Journal of Computer Vision*.

[8] Wexler Y, Simakov D Space-time scene manifolds.

[9] Rav-Acha A, Pritch Y, Lischinski D, Peleg S (2007). Dynamosaicing: mosaicing of dynamic scenes. *IEEE Transactions on Pattern Analysis and Machine Intelligence*.

[10] Frintrop S, Rome E, Christensen HI (2010). Computational visual attention systems and their cognitive foundations: a survey. *ACM Transactions on Applied Perception*.

[11] Borji A, Itti L (2013). State-of-the-art in visual attention modeling. *, IEEE Transactions on Pattern Analysis and Machine Intelligence*.

[12] Ouerhani N, Hugli H Computing visual attention from scene depth.

[13] Itti L, Koch C Comparison of feature combination strategies for saliency-based visual attention systems.

[14] Wolfe JM, Horowitz TS (2004). What attributes guide the deployment of visual attention and how do they do it?. *Nature Reviews Neuroscience*.

[15] Zhang L, Zhang L, Mou X, Zhang D (2011). FSIM: a feature similarity index for image quality assessment. *IEEE Transactions on Image Processing*.

[16] Henriksson L, Hyvärinen A, Vanni S (2009). Representation of cross-frequency spatial phase relationships in human visual cortex. *Journal of Neuroscience*.

[17] Morrone MC, Owens RA (1987). Feature detection from local energy. *Pattern Recognition Letters*.

[18] Kovesi P (2000). Phase congruency: a low-level image invariant. *Psychological Research*.

[19] Penaloza CI, Mae Y, Ohara K Using depth to increase robot visual attention accuracy during tutoring.

[20] Lang C, Nguyen TV, Katti H Depth matters: influence of depth cues on visual saliency.

[21] Howard IP, Rogers BJ (2012). *Perceiving in Depth, Volume 2: Stereoscopic Vision*.

[22] Klaus A, Sormann M, Karner K Segment-based stereo matching using belief propagation and a self-adapting dissimilarity measure.

[23] Bauer R, Delling D, Sanders P (2010). Combining hierarchical and goal-directed speed-up techniques for dijkstra's algorithm. *Journal of Experimental Algorithmics*.

